# Cyclic Vomiting Syndrome-Related Hospitalizations Trends, Comorbidities & Health Care Costs in Children: A Population Based Study

**DOI:** 10.3390/children9010055

**Published:** 2022-01-03

**Authors:** Aravind Thavamani, Krishna Kishore Umapathi, Jasmine Khatana, Sanjay Bhandari, Katja Kovacic, Thangam Venkatesan

**Affiliations:** 1 Division of Pediatric Gastroenterology, Hepatology and Nutrition, UH Rainbow Babies and Children’s Hospital, Case Western Reserve University, Cleveland, OH 44106, USA; 2 Department of Pediatrics, Rush University Medical Center, Chicago, IL 60612, USA; krishnakishoreumapathi@gmail.com; 3 Department of Pediatrics, Metrohealth Medical Center, Cleveland, OH 44106, USA; jkhatana@metrohealth.org; 4 Department of Medicine, Medical College of Wisconsin, Milwaukee, WI 53226, USA; sbhandari@mcw.edu; 5 Division of Pediatric Gastroenterology, Hepatology and Nutrition, Medical College of Wisconsin, Milwaukee, WI 53226, USA; kkovacic@mcw.edu; 6 Division of Gastroenterology and Hepatology, Medical College of Wisconsin, Milwaukee, WI 53226, USA; tvenkate@mcw.edu

**Keywords:** cyclic vomiting syndrome, comorbidities, population-based analysis, health care resource utilization

## Abstract

Aim: To analyze the clinical characteristics, trends in hospitalization and health care resource utilization of pediatric patients with cyclical vomiting syndrome (CVS). Methods: We analyzed the latest 5 Healthcare Cost and Utilization Project-Kids Inpatient Database (HCUP-KID) datasets including years 2003, 2006, 2009, 2012 and 2016 for patients aged 1–20 years with a primary diagnosis of CVS and were compared with Age/gender-matched controls for comorbidities, clinical outcomes, and healthcare resource utilization. Results: A total of 12,396 CVS-related hospitalizations were analyzed. The mean age of CVS patients was 10.4 ± 6.7 years. CVS was associated with dysautonomia (OR: 12.1; CI: 7.0 to 20.8), dyspepsia (OR: 11.9; CI: 8.8 to 16.03), gastroesophageal reflux disease (OR: 6.9; Confidence Interval (CI): 6.4 to 7.5), migraine headaches (OR: 6.8; CI: 5.9 to 7.7) and irritable bowel syndrome (OR: 2.08; CI: 1.2 to 4.3) (all *p* < 0.001). CVS was also associated with increased cannabis use (OR: 5.26, 4.6 to 5.9; *p* < 0.001), anxiety disorder (OR: 3.9; CI: 3.5 to 4.4) and stress reaction (OR: 3.6; CI: 2.06 to 6.3), *p* < 0.001. Mean CVS-related hospitalization costs (inflation adjusted) more than doubled from $3199 in 2003 to $6721 in 2016, incurring $84 million/year in total costs. Conclusion: Hospitalized CVS patients have increased prevalence of DGBIs, dysautonomia, psychiatric conditions and cannabis use compared to non-CVS controls. CVS-related hospitalizations in U.S. is associated with increasing health care costs. Better management of CVS and comorbid conditions is warranted to reduce health care costs and improve outcomes.

## 1. Introduction

Cyclic vomiting syndrome (CVS) is a disorder of gut-brain interaction (DGBI), characterized by recurrent, stereotypical episodes of nausea and vomiting lasting from hours to days [1]. The prevalence rate of pediatric CVS is reported between 1.1 and 6.1% from population-and school-based surveys and data from tertiary care centers [2,3,4,5,6]. A recent population-based study demonstrated a CVS prevalence of 2% among U.S. adults [7]. Comparable data is not available in children although one population-based survey from Ireland reported an incidence rate of pediatric CVS of 3.14 per 100,000 [8]. Although CVS was first described in the literature more than a century ago, the management of CVS has been largely empirical, leading to frequent emergency department (ED) utilization. A previous study of adults and caregivers of children with CVS noted that CVS patients had up to seven emergency department (ED) visits before a diagnosis was confirmed. Further, the diagnosis was not recognized in 88% of CVS cases in the ED [9]. This indicates a lack of awareness and under-recognition of this disorder by health care providers [9]. Population-based studies among adults also demonstrate that CVS patients have a high number of comorbidities and that these patients incur substantial health care costs and utilization [10]. 

Most studies in CVS are from specialized, tertiary referral centers and there is a paucity of data on the national trends in pediatric CVS in the U.S. We therefore aimed to analyze the clinical characteristics, trends in hospitalization and health care resource utilization of pediatric patients with cyclical vomiting syndrome (CVS) using a large nationwide database. 

## 2. Materials and Methods

We analyzed data from the Healthcare Cost and Utilization Project Kids Inpatient Database (HCUP-KID). KID database is a nationwide collection of data on hospitalized patients up to 20 years of age, and has an aggregated collection of stratified random samples across the U.S. As KID datasets are released once every 3 years, we analyzed the latest 5 KID datasets corresponding to years 2003, 2006, 2009, 2012 and 2016. Each record in the database represents an inpatient discharge encounter and has up to 30 diagnostic and 15 procedural codes all of which are classified as per International Classification of Diseases (ICD)-9, except for KID 2016 which employed ICD-10 for diagnosis and coding. 

### 2.1. Patient Population

In the present study, we identified CVS patients using ICD-9 code (536.2) and ICD-10 codes (G43.A0 or G43.A1 or R11.15) and only included patients who had a primary diagnosis of CVS at the time of discharge. Among patients who did not have a diagnosis of CVS in any of the primary or the secondary diagnosis columns in the database, we selected age and gender-matched controls in a ratio of 1:5. We excluded patients who were less than one year of age, as this age group were comprised mostly of normal healthy newborns and infants. Further, we also excluded conditions which can present with recurrent vomiting and mimic CVS such as hydronephrosis, malrotation, eosinophilic esophagitis, rumination syndrome, gastroparesis, hydrocephalus, metabolic disorders including acute intermittent porphyria, disorders of fatty acid oxidation, urea cycle defects, disorders of mitochondrial energy metabolism, and disorders of organic and amino acid metabolism. In order to accurately capture cases with CVS, we also excluded patients with a concomitant diagnosis of pregnancy-associated emesis, post-surgical and post-operative nausea and vomiting as well as psychogenic causes of vomiting. We subsequently analyzed the discharge records of these patients for other comorbid conditions known to be associated with CVS, which were selected a priori and included for analysis using ICD-9 & 10 codes. 

### 2.2. Study Variables

We analyzed various demographic data: age, sex, race, type of insurance and hospitalization data. Race was classified as White, African American (AA), Hispanic and Other. Insurance data was derived from the primary payer information and categorized as Public (Medicare & Medicaid), Private (commercial insurance or health maintenance organization) and Uninsured or Self-pay/others. Comparisons between the CVS and non-CVS groups included demographics, clinical characteristics, outcomes and hospital costs. Discharge weights provided in the datasets were used in the calculation of national estimates. We calculated the total hospitalization costs from total hospital charges using the cost to charge ratio (CCR) variable provided in the KID datasets. As per the data user agreement of HCUP project, any column with less than 10 patients were not reported. ICD diagnostic and procedure codes of all the variables are described in Supplementary Table S1.

### 2.3. Statistical Analysis

National trends were calculated after applying the population weights provided in the KID database. The basic demographic profile of the population was reported as numbers and percentages. Chi-Square test was used to analyze the categorical data across different groups. Mann-Whitney U test was used for quantitative variable including length of stay (LOS) and total charge and results were described as mean and SD. Multivariate logistic regression model was used to analyze and compare the adjusted odds ratio (OR) of various comorbid conditions associated with CVS hospitalization. We included all the factors associated with outcome on univariate analysis with a *p* value of <0.2. All adjusted ORs were considered significant if the 95% confidence interval did not include 1.00. Trend analysis was performed using weighted least square regression analysis. All data analyses were performed with SPSS 24 (IBM Corporation, Armonk, NY, USA).

## 3. Results

### 3.1. Demographics and Clinical Characteristics of CVS and Non-CVS Patients

The demographics of our study population are displayed in Table 1. The mean age of patients with CVS was 10.5 ± 6.7 years and was comparable to 10.6 ± 7.8 in the control population (*p* = 0.21). More than half of the patients with CVS were female (57%). Compared to the non-CVS group, CVS patients were predominantly White (49% vs. 42%), had private insurance (51% vs. 43%) and received care in urban teaching hospitals (64% vs. 59%) (*p* < 0.001). The CVS group had a higher proportion comorbidity including irritable bowel syndrome (1% vs. 0.1%), dysautonomia (0.6% vs. <0.1%), gastroesophageal reflux disease (GERD) (14.1% vs. 2.2%), and dyspepsia (1.9% vs. 0.1%) compared to the non-CVS group (all *p*-values < 0.001). The proportion of CVS patients with migraine headache was significantly higher compared to the non-CVS population (5.6% vs. 0.8%) (*p* < 0.001). There was no significant association between obesity and CVS (*p* = 0.16). While alcohol use was less prevalent among patients with CVS, these patients tended to be significantly older (18.5 ± 1.4 years) compared to CVS patients without any alcohol use (10.4 ± 6.7) (*p* < 0.001). Similarly, CVS patients using cannabis were significantly older (18.5 ± 1.4 years) compared to CVS patients without cannabis use (9.9 ± 6.6 years), *p* < 0.001. 

### 3.2. Trends in CVS-Related Hospitalization

**Figure 1:**
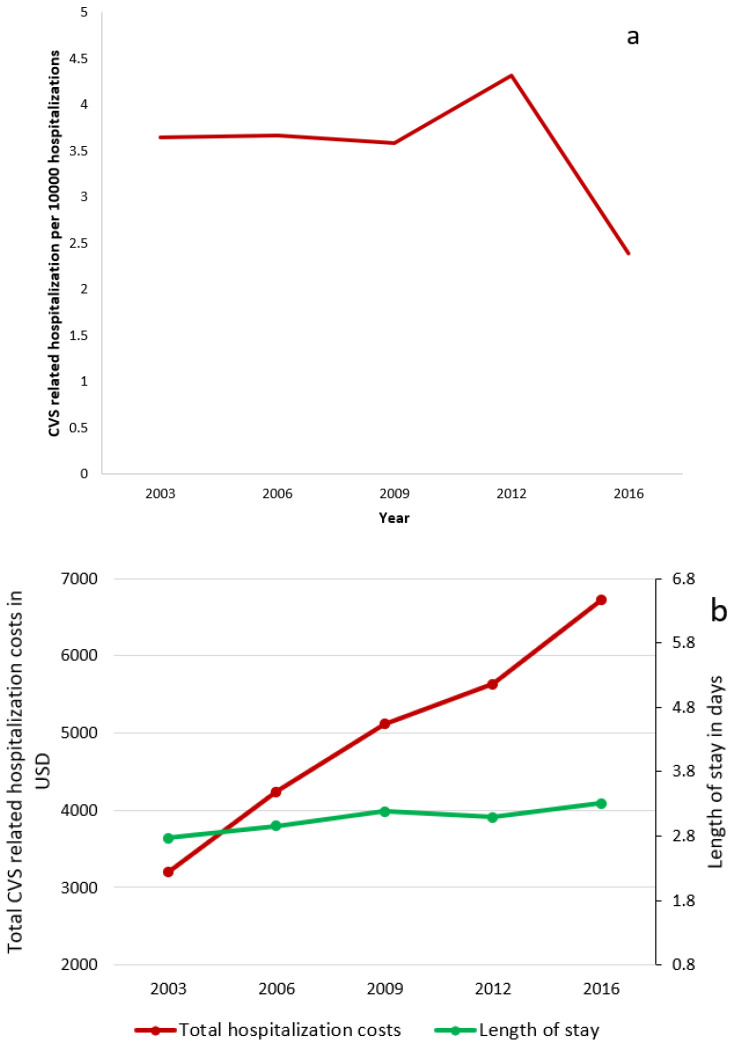
(**a**) Trend of CVS-related hospitalization rate between 2003 and 2016 using KID database; (**b**) Trend of hospitalization costs and length of stay for CVS related hospitalization.

The total number of CVS-related hospitalizations was 12,396 compared to 60,735 for the age and gender-matched control subjects. The overall pediatric hospitalization rate during the study period was 3.54 per 10,000 hospitalizations. The rate of CVS-related hospitalization was 3.64 per 10,000 in 2003 which increased to a peak of 4.13 per 10,000 in 2012 followed by a decrease to 2.4 per 10,000 hospitalizations. Although the rate of hospitalization was fluctuating, there was no significant change in the trend analysis, *p* = 0.14 (Figure 1a).

### 3.3. GI Procedures, Costs and Length of Hospital Stay

Patients with CVS underwent more gastrointestinal procedures during their hospitalizations (Table 2). Esophagogastroduodenoscopy (EGD) (10.4% vs. 0.8%) and colonoscopy (1.1% vs. 0.3%) were significantly higher among CVS patients compared to non-CVS controls (*p* < 0.001). Nuclear medicine scan/radioisotope studies were done only in a fraction of CVS patients (0.3%) during the hospital admission, but still statistically higher than in the control population (*p* < 0.001). More CVS patients were discharged against medical advice compared to the non-CVS control population (1.3% vs. 0.3%) (*p* < 0.001). Mortality rate was also significantly lower in CVS patients (<0.1%) than in controls (0.4%) (*p* < 0.001) (Table 2). The length of stay (LOS) and total costs were lower in patients with CVS than in the control population (Table 2). However, analysis of trends between the years 2003 and 2016 showed a significant increase in total hospitalization costs and length of stay over time (*p* < 0.01) (Figure 1b).

### 3.4. Factors Associated with CVS on Multivariate Analysis

On multivariate analysis, AA and Hispanic race were less likely to be associated with CVS when compared to White race: AA vs. White OR = 0.87 (CI: 0.81 to 0.93, *p* < 0.001) and Hispanic vs. White: OR = 0.72 (CI: 0.67 to 0.77, *p* < 0.001). Similarly, patients with CVS had higher odds of having private insurance: OR = 1.25 (CI: 1.2 to 1.3, *p* < 0.001). Further, hospitals with larger bed size had lower odds of CVS hospitalizations when compared to hospitals with smaller bed size: OR = 0.79 (CI: 0.75 to 0.85, *p* < 0.001). Similarly, urban hospitals were less likely to be associated with CVS hospitalizations compared to rural hospitals: Urban non-teaching vs. rural hospital OR = 0.63 (0.58 to 0.67, *p* < 0.001) and urban teaching vs. rural hospital: OR = 0.81 (0.75 to 0.86, *p* < 0.001) (Table 3). 

CVS-related hospitalizations had higher odds of being associated with comorbid conditions including dyspepsia, dysautonomia, migraine headache, GERD and Irritable bowel syndrome (IBS) (Table 3). The CVS group also had an increased odds of associated cannabis use: OR = 5.2, (CI: 4.64 to 5.96, *p* < 0.001) and decreased alcohol use than controls: OR = 0.31 (CI: 0.25 to 0.41, *p* < 0.001). CVS was also more likely associated with an anxiety disorder: OR = 3.9 (CI: 3.5 to 4.4, *p* < 0.001) and stress reaction: OR = 3.6 (CI: 2.06 to 6.29, *p* < 0.001), compared to the non-CVS control group (Table 3).

## 4. Discussion

This is the largest population-based study analyzing hospitalization trends, clinical characteristics and comorbidities associated with CVS in the pediatric age group. Girls are more commonly affected than boys and the majority of hospitalized CVS patients are white. Similar to studies in adults, pediatric patients with CVS had a higher degree of comorbidities including dyspepsia, GERD, migraines and dysautonomia [11]. Hospitalized patients with CVS had an increased prevalence of anxiety disorders compared to the control population. 

Our study revealed that the rate of pediatric CVS-related hospitalizations has remained overall stable over time except in 2016 in which the rate of hospitalization had decreased, this could be related to the transition from ICD-9 to ICD-10 codes. In our study, the overall proportion of IBS among CVS patients was 1% compared 0.1% among the control population. The odds of having associated IBS were more than two times higher among patients with CVS (Table 3). This is in contrast to the study by Chelimsky et al. of pediatric patients with CVS and migraine, in which they did not find any significant association between IBS and CVS [12]. However, various adult studies have shown a clear association between IBS and CVS with prevalence rates ranging from 5% to 32% among adults [10,13,14,15]. The overall low rate of IBS compared to its prevalence in the general population, is because this only represents patients with CVS who were hospitalized. 

We found that migraine headache was increasingly more common among patients with CVS (5.7% vs. 0.8%) consistent with the existing literature. A previous population-based study performed in a pediatric cohort in Scotland showed that almost 21% of CVS patients had migraines, though this was limited by the small sample size of 34 patients. A similar study among 24 Turkish pediatric patients showed that migraine was prevalent in 25% (6/24) of the CVS patients and almost 29% of the patients had a strong family history of migraines [3]. A study of CVS patients in Ireland showed that prevalence of migraine headache and non-migraine headaches were 2.4% and 5% respectively among newly diagnosed cases [8]. The highest reported prevalence rate of migraine associated CVS was 82% by Li et al. [16]. However, in their study, they used a family history of migraine or subsequent development of migraine to define migraine-related CVS, which could have contributed to this increased prevalence of migraine compared to other studies [16]. 

Dysautonomia was more common in pediatric age group (0.6%) when compared to a similar adult (0.3%) study10. In a single center study, orthostatic intolerance was reported in about 10 out of 21 (47%) patients with CVS [12]. Further, in a study by Ojha et al. of patients with postural orthostatic tachycardia syndrome (POTS), 12/20 patients had recurrent nausea/vomiting although it is unclear how many may have represented CVS [17]. A prospective study in adults showed that POTS was present in 85% of patients [18].

The prevalence of cannabis use was 6%, which is significantly lower than the 14% reported among adult population-based studies on CVS [10]. This would be expected given that this study was performed mostly in children. CVS patients with cannabis use were significantly older than CVS patients without cannabis use and we report a greater than 5 times increased odds of associated cannabis use among CVS patients. This is important given the increasing use of cannabis amongst adolescents and also increasing legalization and perception about its use in the US. Choung et al. found that cannabis use was more prevalent among CVS patients compared to those with functional vomiting (79% vs. 21%) [19]. Similarly, a survey of adult patients with CVS showed that almost 81% had used cannabis for its therapeutic effects [20]. Cannabis has antiemetic properties demonstrated by both animal and human studies. It acts through the cannabinoid receptor type 1 (CB1R), which is densely distributed in in areas of the brain that are associated with emesis. Cannabis is thought to have a biphasic effect with low doses exerting antiemetic effects while higher doses paradoxically result in hyperemesis. The cause for this is not clear but may be due to increasing potency of cannabis products and subsequent downregulation of CB1R [21,22,23]. Whether cannabinoid hyperemesis syndrome is a subset of CVS or an entirely separate entity remains unclear. Further assessment of a causal relationship with cannabis cessation is often confounded by the self-limiting nature of these episodes, and the reluctance of patients to stop using cannabis due to its reported beneficial effects, thus making the distinction difficult to establish in clinical practice. 

Anxiety was more commonly seen in pediatric CVS patients (7.8%) compared to the control population (1.8%) and the proportion was significantly higher in the adolescents (12.8%). In a study of 40 CVS patients, Tarbell et al. reported a prevalence of anxiety of 15% and 27% based on parent-proxy and self-report respectively [24]. They also demonstrated that anxiety was an independent predictor of health-related quality of life. The prevalence of anxiety among adolescent and youth population with CVS varies from 15–64% across different studies [24,25,26]. This is considerably higher compared to our study results and possible reasons for this discrepancy might be related to diagnostic coding and the use of only hospitalized patients from a database in our study. Nevertheless, we found that the prevalence of anxiety increased from 5.3% in 2003 to more than 26.8% in 2016, indicating an increasingly strong association between anxiety and CVS. In our study, pediatric CVS patients had a 4 times higher odds of an associated anxiety disorder compared to 2.5 times in the adult population [10]. CVS is often precipitated by psychological or physiological stress. Although only a minority of patients (0.2%) had a diagnosis of stress reactions, CVS patients also had increased odds of associated stress reaction compared to non-CVS controls.

The length of hospital stay was shorter in CVS compared to controls. Total mean hospitalization cost was 84,430,976 USD/year but lower compared to controls and is consistent with data in adults [10]. These numbers may reflect a heterogenous control population with patients with complex disorders requiring prolonged hospitalizations, incurring higher costs compared to CVS hospitalizations. However, the total CVS-related hospitalization costs (inflation adjusted) more than doubled from 3199 USD in 2003 to 6721 USD in 2016 (Figure 1b). The mean length of stay also increased marginally from 2.77 days in 2003 to 3.31 days in 2016. The increase in cost could be due to more extensive use of diagnostic tests. It has been shown that such a strategy increased costs but did not result in a change in diagnosis or management in most patients [27]. 

Our study has various limitations. This was a retrospective study and thus associated with the inherent limitations of such studies. The database relies on the accuracy of coding to diagnose CVS. Although ICD-9 does not have a unique diagnostic code for CVS, we employed methods similar to published adult study to capture CVS patients using ICD-9 code for persistent emesis while excluding comprehensive list of diagnoses which may mimic10. Coding errors are inevitable in such large databases and should be considered while interpreting the results. Information regarding outpatient visits and medications administered during the hospitalizations are not available. Each case represents a hospital discharge event and not an individual patient. Therefore, repeat hospitalizations were not captured for the same patients using this database and it may thus overestimate the incidence of CVS-related hospitalizations. Similarly, the percentage of comorbid conditions could be vastly overestimated for the same reason. Information regarding the readmission rates was not available for analysis. Despite these limitations, our study is the largest population-based study to date that reports on trends in CVS-related hospitalizations, clinical characteristics and costs in a cohort of children hospitalized for CVS.

## 5. Conclusions

In summary, CVS patients also have a high burden of other DGBIs, anxiety and cannabis use which need to be addressed in the management of these patients. Further, pediatric CVS incurs a significant health care burden. The costs of care are substantial and have increased over time. These costs do not include other challenging aspects of CVS care such as emergency department use, days lost from school and work absenteeism (caregiver). 

## Figures and Tables

**Table 1:** Comparison of demographics and clinical characteristics of the CVS and non-CVS patients.

Demographics	CVS N = 12,396	Controls N = 60,735	*p* Value
Age	10.51 ± 6.7	10.61 ± 7.8	0.15
Sex Male Female	5352 (43.2%) 7044 (56.8%)	26,115 (43%) 34,619 (57%)	0.73
Race White African American Hispanic Others	6117 (49.3%) 1481 (11.9%) 1628 (13.1%) 3170 (25.6%)	25,738 (42.4%) 8522 (14%) 11,383 (18.7%) 15,092 (24.9%)	<0.001
Insurance Public Private Self-pay/Uninsured/others	4989 (40.2%) 6336 (51.1%) 1071 (8.7%)	29,045 (47.8%) 26,256 (43.2%) 5434 (9%)	<0.001
Bed Size of Hospital Small Medium Large	1677 (13.8%) 3509 (29%) 6926 (57.2%)	7403 (12.4%) 15,247 (25.6%) 36,843 (61.9%)	<0.001
Location/Teaching status Rural Urban—Nonteaching Urban Teaching	1495 (12.3%) 2866 (23.7%) 7752 (64%)	6391 (10.7%) 17,912 (30.1%) 35,191 (59.3%)	<0.001
Region of Hospital Northeast Midwest South West	2519 (20.3%) 2803 (22.6%) 4185 (33.8%) 2889 (23.3%)	10,347 (17%) 13,911 (22.9%) 23,672 (39%) 12,805 (21.1%)	<0.001
Elective hospitalizations	1249 (10.1%)	10,457 (17.3%)	<0.001
Comorbid Conditions			
Irritable bowel syndrome	124 (1%)	83 (0.1%)	<0.001
Migraine	697 (5.6%)	457 (0.8%)	<0.001
Dysautonomia	69 (0.6%)	19 (<0.1%)	<0.001
Gastroesophageal reflux disease	1747 (14.1%)	1344 (2.2%)	<0.001
Obesity	255 (2.1%)	1122 (1.8%)	0.16
Dyspepsia	238 (1.9%)	66 (0.1%)	<0.001
Substance Abuse			
Narcotic use	64 (0.5%)	255 (0.4%)	0.13
Alcohol use	58 (0.7%)	575 (0.9%)	0.006
Cannabis use	745 (6%)	849 (1.4%)	<0.001
Smoking	531 (4.3%)	1589 (2.6%)	<0.001
Mental Health Disorders			
Depression	532 (4.3%)	2461(4.1%)	0.22
Anxiety	967 (7.8%)	1119 (1.8%)	<0.001
Adjustment disorder	173 (1.4%)	839 (1.4%)	0.90
Stress reaction	25 (0.2%)	38 (0.1%)	<0.001
Post-traumatic stress disorder	81 (0.7%)	534 (0.9%)	0.01
Bipolar Disorder	140 (1.1%)	1042 (1.7%)	<0.001

**Table 2:** Comparison of Gastrointestinal procedures and hospital outcomes between the groups.

Procedures/Outcomes	CVS N = 12,624	Controls N = 60,735	*p* Value
Esophagogastroduodenoscopy	1288 (10.4%)	478 (0.8%)	<0.001
Colonoscopy	135 (1.1%)	208 (0.3%)	<0.001
Length of hospital stay (days)	3.03 ± 0.03	3.79 ± 0.03	<0.001
Total Charges (in dollars)	14,225 ± 197	21,181 ± 272	<0.001
Total cost	4974 ± 71	6925 ± 88	<0.001
Discharge Routine Against Medical Advice	11,581 (93.4%) 155 (1.3%)	56,910 (93.7%) 191 (0.3%)	0.13 <0.001
Mortality	IS (<0.1%)	144 (0.4%)	<0.001

**Table 3:** Multivariate logistic regression model demonstrating the association between various demographic factors, comorbid conditions and CVS hospitalizations.

Parameters	OR	95% Confidence Interval	*p* Value
Race White African American vs. White Hispanic vs. White Others vs. White	Ref 0.87 0.72 0.95	Ref 0.81 to 0.93 0.67 to 0.76 0.90 to 1.004	Ref <0.001 <0.001 0.068
Insurance Public Private vs. public Self-pay/Insured vs. public	Ref 1.25 1.05	Ref 1.19 to 1.31 0.97 to 1.14	Ref <0.001 0.17
Hospital Bed Size Small Medium vs. Small Large vs. Small	Ref 1.005 0.79	Ref 0.93 to 1.07 0.75 to 0.85	Ref 0.19 <0.001
Teaching Status Rural Urban nonteaching vs. rural Urban teaching vs. rural	Ref 0.63 0.81	Ref 0.58 to 0.67 0.75 to 0.86	Ref <0.001 <0.001
Location Northeast Midwest vs. Northeast South vs. Northeast West vs. Northeast	Ref 0.76 0.79 1.03	Ref 0.71 to 0.82 0.75 to 0.84 0.96 to 1.1	Ref <0.001 <0.001 0.38
Year 2003 2006 vs. 2003 2009 vs. 2003 2012 vs. 2003 2016 vs. 2003	Ref 0.97 0.88 0.89 0.48	Ref 0.91 to 1.03 0.83 to 0.94 0.89 to 1.01 0.45 to 0.52	Ref 0.35 <0.001 0.14 <0.001
Irritable bowel syndrome	2.08	1.2 to 4.31	<0.001
Migraine	6.79	5.96 to 7.74	<0.001
Dysautonomia	12.08	7.003 to 20.844	<0.001
Gastroesophageal reflux disease	6.9	6.4 to 7.51	<0.001
Dyspepsia	11.89	8.82 to 16.03	<0.001
Alcohol use	0.29	0.22 to 0.38	<0.001
Cannabis use	5.26	4.64 to 5.96	<0.001
Smoking	1.13	1.003 to 1.27	0.04
Anxiety	3.99	3.59 to 4.43	<0.001
Stress Reaction	3.60	2.06 to 6.29	<0.001
Post-traumatic stress disorder	0.51	0.39 to 0.67	0.001
Bipolar Disorder	0.39	0.32 to 0.48	<0.001

## Data Availability

All data analyzed were part of publicly available HCUP databases.

## Supplementary Materials

The following are available online at https://www.mdpi.com/article/10.3390/children9010055/s1, Table S1: ICD-9 and 10 codes.
