# Supplementary material for: Cyclic Vomiting Syndrome-Related Hospitalizations Trends, Comorbidities & Health Care Costs in Children: A Population Based Study
Source: Children (Basel). 2022 Jan 3;9(1):55. doi: 10.3390/children9010055 (PMC8774277; doi:10.3390/children9010055)
Supplement: Supplementary file 1 [file children-09-00055-s001.zip › children-1505142-supplementary.pdf]

Supplementary

Table S1. ICD 9 and 10 codes.

Diagnosis	ICD-9	ICD-10
Cyclical Vomiting Syndrome	536.2	G43.A0;G43.A1;R11.15
Gastroparesis	536.3	K31.84
Irritable bowel syndrome	564.1	K58.x
Migraine	346.xx	G43.x
Dysautonomia	337.9	G901, G908, G909
Gastroesophageal reflux disease	530.81	K21.x
Obesity	278.0x	E66.x
Dyspepsia	536.8	K30
Narcotic use	304.0x,304.7x, 305.5x	F11.x
Alcohol use	303.xx, 305.0x	F10.x
Cannabis use	304.3x, 305.2x	F12.x
Smoking	305.1, V1582,989.84	F17.x, Z120, T65.x
Dyspepsia	536.8	K30
Depression	292.2x, 293.3x, 311	F32.x
Anxiety	300.00, 300.02, 300.09	F41.x
Adjustment disorder	309.0, 309.2 to 309.9	F43.2x
Stress reaction	308.3, 308.4, 308.9	F43.0, F43.8, F43.9
Post traumatic disorder	309.81	F43.1x
Bipolar disorder	296.0x, 296.4, 296.5, 296.6	F31.x

X denotes all subcodes within the ICD parent code.
